# Supplementary material for: Dialogic gathering of films. Promoting meaningful online interactions during COVID-19 confinement
Source: PLoS One. 2021 Jul 9;16(7):e0254132. doi: 10.1371/journal.pone.0254132 (PMC8270149; doi:10.1371/journal.pone.0254132)
Supplement: S1 Questionnaire — (PDF) [file pone.0254132.s001.pdf]

CUESTIONARIO.

TERTULIA DIALÓGICA DE PELÍCULAS

Sección 1. Tertulia Dialógica de Películas

1. **Género**
2. **Edad**
3. **Ciudad de residencia**
4. **Nacionalidad**
5. **Ámbito profesional**
6. **¿Cómo has accedido a la Tertulias Dialógicas de Películas?**
7. **¿Qué te ha llevado a participar en las Tertulias Dialógicas de Películas?**
8. **¿Se te ha exigido alguna condición para poder participar?**

- SÍ (Explica cuál en la siguiente pregunta)
- No

9. **En caso de respuesta afirmativa a la anterior pregunta, ¿Qué condición se te ha exigido?**

10. ¿En qué debates has participado?

o	Virus
o	Documental: Claudio Monteverdi
o	Ciudadano Kane
o	Cotton Club
o	Vivir
o	Baahubali I
o	La Peste
o	Las mil y una noches
o	La hija de Brest
o	Romero
o	Y los violines dejaron de sonar
o	Florence Nightingale
o	Ulises
o	Oliver Twist
o	Ready Player One
o	Documental: Inteligencia Artificial. Nuestra mejor amiga.
o	Sócrates
o	Lawerence of Arabia
o	Documental: ¿Por qué la belleza importa?
o	Código Enigma
o	Opera: Fidelio (Beethoven)
o	Casablanca
o	Doctor Zhivago
o	Documental: Neurociencia Conferenceia del Dr. Leone
o	Selma
o	Documental: Vi(5G)ilados
o	Los Nibelungos (1ª parte). La muerte de Sigfrido.

Sección 2. Descripción de las Tertulias Dialógicas de Películas

11. ¿Siempre introduce los debates la misma persona o cada vez una persona distinta?

12. Valora la introducción

Pobre 1 2 3 4 Muy Buena

13. Explica por qué, si quieres

14. ¿Qué formato tiene el debate? ¿Cómo se interviene?

15. **Valora el formato**

Pobre 1 2 3 4 Muy Bueno

16. **¿Quiénes intervienen en el debate? Describe la diversidad que existe en los debates**

17. **Valora la diversidad**

Poca 1 2 3 4 Mucha

18. **¿Quiénes intervienen en el debate? Comenta sobre la proporción de gente que interviene en cada sesión**

19. **Valora la proporción de gente que interviene en cada sesión**

Poca 1 2 3 4 Mucha

20. **Valora los tiempos en el debate: Inicio y fin, puntualidad, duración, extensión de las intervenciones...**

21. **Valora los tiempos en el debate**

Poco adecuados 1 2 3 4 Muy adecuados

22. **¿Te parece bien que se elijan las películas con las que se generen debates más plurales y ricos culturalmente?**

23. **Valora la selección de las películas**

Poco adecuada 1 2 3 4 Muy adecuada

24. **¿Qué tipos de conocimientos se comparten en los debates?**

25. **Valora el nivel intelectual de los debates**

Muy bajo 1 2 3 4 Muy alto

26. **¿Crees que el debate aporta conocimientos intelectuales, culturales y científicos de calidad que no aparecen en la película y son importantes? ¿Qué te lo hace pensar?**

27. **Valora los conocimientos que complementan el debate**

Muy bajos 1 2 3 4 Muy altos

28. ¿Alguien os ha impedido a ti o a algún compañero/a expresar vuestra opinión?

29. Valorar la libertad para expresar una opinión en el debate

Poca libertad 1 2 3 4 Mucha libertad

30. ¿Alguien ha descalificado tu aportación o la de un compañero/a?

31. Valora la frecuencia con la que se respetan todas las aportaciones

Nunca 1 2 3 4 Siempre

Sección 3. Impacto de las Tertulias Dialógicas de Películas

32. **¿Qué película o debates te han generado más reflexiones? ¿Por qué crees?**

33. **¿Qué películas o debates has compartido con otras personas?**

o	Virus
o	Documental: Claudio Monteverdi
o	Ciudadano Kane
o	Cotton Club
o	Vivir
o	Baahubali I
o	La Peste
o	Las mil y una noches
o	La hija de Brest
o	Romero
o	Y los violines dejaron de sonar
o	Florence Nightingale
o	Ulises
o	Oliver Twist
o	Ready Player One
o	Documental: Inteligencia Artificial. Nuestra mejor amiga.
o	Sócrates
o	Lawerence of Arabia
o	Documental: ¿Por qué la belleza importa?
o	Código Enigma
o	Opera: Fidelio (Beethoven)
o	Casablanca
o	Doctor Zhivago
o	Documental: Neurociencia Conferenceia del Dr. Leone
o	Selma
o	Documental: Vi(5G)ilados
o	Los Nibelungos (1ª parte). La muerte de Sigfrido.

34. **¿En qué ámbito o contexto has compartido las películas/debates con otras personas?**

35. **¿Por qué motivos has compartido las Tertulias Dialógicas de Películas?**

36. **¿Has vuelto a ver alguna de las películas en otro espacio?**

37. **¿En qué te ha ayudado participar en las Tertulias Dialógicas de Películas a nivel profesional?**

38. **Valora cuánto te ha ayudado participar en las Tertulias Dialógicas de Películas a nivel profesional**

Poco 1 2 3 4 Mucho

39. **¿Crees que participar en las Tertulias Dialógicas de Películas te ayudará a nivel profesional en el futuro?**
40. **¿Puedes dar algún detalle sobre cómo te ha servido o servirá en tu profesión?**
41. **Valora cuánto crees que te puede ayudar participar en las Tertulias Dialógicas de Películas a nivel profesional**

    Poco 1 2 3 4 Mucho

42. **¿En qué te ha ayudado participar en las Tertulias Dialógicas de Películas a nivel personal?**
43. **Valora cuánto te ha ayudado participar en las Tertulias Dialógicas de Películas a nivel personal**

Poco 1 2 3 4 Mucho

44. **¿Cómo te ha ayudado participar en las Tertulias Dialógicas de Películas a lidiar con la situación social generada por el COVID-19?**
45. **Valora cuánto te ha ayudado participar en las Tertulias Dialógicas de Películas a lidiar con la situación del COVID-19**

    Poco 1 2 3 4 Mucho

46. **¿En qué medida ha impactado tu implicación para contribuir a la situación del COVID-19?**
47. **Valora hasta qué punto, en comparación con otras actividades, te han animado las Tertulias Dialógicas de Películas a trabajar contra el COVID-19**

Poco 1 2 3 4 Mucho

48. ¿Crees que las sesiones te aportan igual, intervengas o no?

49. Valora cuánto te aportan las Tertulias Dialógicas de Películas

Poco 1 2 3 4 Mucho

50. ¿Tienes alguna otra aportación que quieras compartir?
