# Supplementary material for: Dialogic gathering of films. Promoting meaningful online interactions during COVID-19 confinement
Source: PLoS One. 2021 Jul 9;16(7):e0254132. doi: 10.1371/journal.pone.0254132 (PMC8270149; doi:10.1371/journal.pone.0254132)
Supplement: S2 Questionnaire — (PDF) [file pone.0254132.s002.pdf]

Questionnaire

Dialogic Gathering of Films

Section 1. Dialogic Gathering of Films

1. **Gender**
2. **Age**
3. **City of residence**
4. **Nationality**
5. **Professional field**
6. **How did you learn about the Dialogical Gathering of Films?**
7. **What led you to participate in the Dialogical Gathering of Films?**
8. **Are there any requirements to participate?**

- o Yes (Explain which one in the next question)
- o No

9. **If you answered yes to the above question, please explain the requirements asked from you.**

10. In which debates have you participated?

○	Virus
○	Documentary Film: Claudio Monteverdi
○	Citizen Kane
○	Cotton Club
○	To live
○	Baahubali I
○	The Plague
○	Arabian Nights
○	La fille de Brest
○	Romero
○	And the Violins Stopped Playing
○	Florence Nightingale
○	Ulises
○	Oliver Twist
○	Ready Player One
○	Documentary Film: Artificial Intelligence. Our best friend
○	Socrates
○	Lawerence of Arabia
○	Documentary Film: Why Beauty Matters
○	The Imitation Game
○	Opera: Fidelio (Beethoven)
○	Casablanca
○	Doctor Zhivago
○	Documentary film: Neuroscience Conference from Dr. Leone
○	Selma
○	Documentary Film: Vi(5G)ilados
○	The Nibelungs (1st part). The death of Siegfried

Section 2. Description of the Dialogic Gathering of Films.

11. **Does the same person always introduce the discussions or is there a different person each time?**
12. **Rate the introduction**

    Poor 1 2 3 4 Very Good
13. **Explain why, if you want to**
14. **What is the format of the debate and how do you intervene?**
15. **Rate the format**

    Poor 1 2 3 4 Very Good
16. **Who is involved in the debate? Describe the diversity that exists in the debates**
17. **Rate the diversity**

    Little 1 2 3 4 A lot
18. **Who is involved in the debate? Comment on the proportion of people who intervene in each session**
19. **Value the proportion of people involved in each session**

    Little 1 2 3 4 A lot
20. **Evaluates the times in the debate: Start and end, punctuality, duration, length of interventions...**
21. **Evaluate the time spent in the debate**

    Not very suitable 1 2 3 4 Very suitable

22. Do you think it is right to choose the films that generate the most pluralistic and culturally rich discussions?

23. Rate the selection of the films

Not very suitable 1 2 3 4 Very suitable

24. Which knowledge is shared in the discussions?

25. Assess the intellectual level of the debates

Very low 1 2 3 4 Very high

26. Do you think the debate provides intellectual, cultural and scientific knowledge of quality that that does not appear in the film and is important?? What makes you think so?

27. Value the knowledge that complements the debate

Very low 1 2 3 4 Very high

28. Has anyone stopped you or another participant from expressing an opinion?

29. Value the freedom to express an opinion in the debate

Little freedom 1 2 3 4 Much freedom

30. Has anyone disqualified your contribution or that of another participant?

31. Value the frequency with which all contributions are respected

Never 1 2 3 4 Always

Section 3 Impact of Dialogic Gatherings of Films

32. Which film or debates have generated the most reflections in you? Why do you think this is?

33. Which films or debates have you shared with others

○	Virus
○	Documentary Film: Claudio Monteverdi
○	Citizen Kane
○	Cotton Club
○	To live
○	Baahubali I
○	The Plague
○	Arabian Nights
○	La fille de Brest
○	Romero
○	And the Violins Stopped Playing
○	Florence Nightingale
○	Ulises
○	Oliver Twist
○	Ready Player One
○	Documentary Film: Artificial Intelligence. Our best friend
○	Socrates
○	Lawerence of Arabia
○	Documentary Film: Why Beauty Matters
○	The Imitation Game
○	Opera: Fidelio (Beethoven)
○	Casablanca
○	Doctor Zhivago
○	Documentary film: Neuroscience Conference from Dr. Leone
○	Selma
○	Documentary Film: Vi(5G)ilados
○	The Nibelungs (1st part). The death of Siegfried

34. In what area or context have you shared the films/discussions with others?

35. For what reasons have you shared the Dialogic Gathering of Films?

36. Have you seen any of the films in another space?

37. **How has participating in the Dialogic Gathering of Films helped you professionally?**

38. **Rate how much participating in the Dialogic Gathering of Films has helped you at a professional level**

    A little 1 2 3 4 A lot

39. **Do you think participating in the Dialogic Gathering of Films will help you professionally in the future?**

40. **Can you give some details about how it has served or will serve you in your profession?**

41. **Rate how much you think participating in Dialogic Gathering of Films can help you professionally**

    A little 1 2 3 4 A lot

42. **How has participating in Dialogic Gathering of Films helped you on a personal level?**

43. **Rate how much has participating in the Dialogic Gathering of Films helped you on a personal level?**

    A little 1 2 3 4 A lot

44. **How has participating in the Dialogic Gathering of Films helped you to deal with the social situation generated by the COVID-19 pandemic?**

45. **Rate how much has participating in the Dialogical Gathering of Films helped you to deal with the situation generated by the COVID-19 pandemic**

    Poco 1 2 3 4 Mucho

46. **To what extent has your involvement impacted you on contributing to the COVID-19 situation?**

47. Rate the extent to what, in comparison with other activities, you have been encouraged by the Dialogic Gathering of Films to work against COVID-19

A little 1 2 3 4 A lot

48. Do you think the sessions bring you the same benefits whether you intervene or not?

49. Rate how much the Dialogic Gathering of Films benefits you

A little 1 2 3 4 A lot

50. **Do you have any other contribution you would like to share?**
