# Supplementary material for: Dialogic gathering of films. Promoting meaningful online interactions during COVID-19 confinement
Source: PLoS One. 2021 Jul 9;16(7):e0254132. doi: 10.1371/journal.pone.0254132 (PMC8270149; doi:10.1371/journal.pone.0254132)

Results of the closed-ended data from the Likert-scale questionnaire

12. Rate the introduction

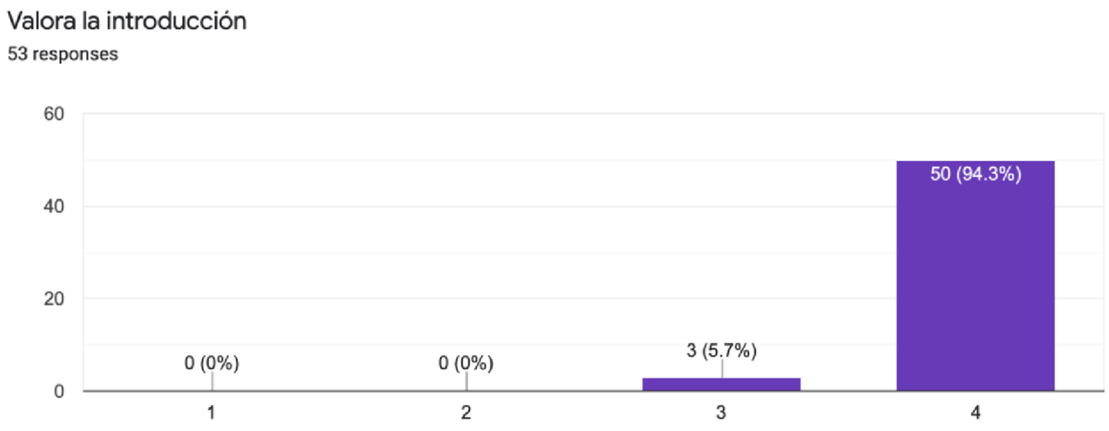

15. Rate the format

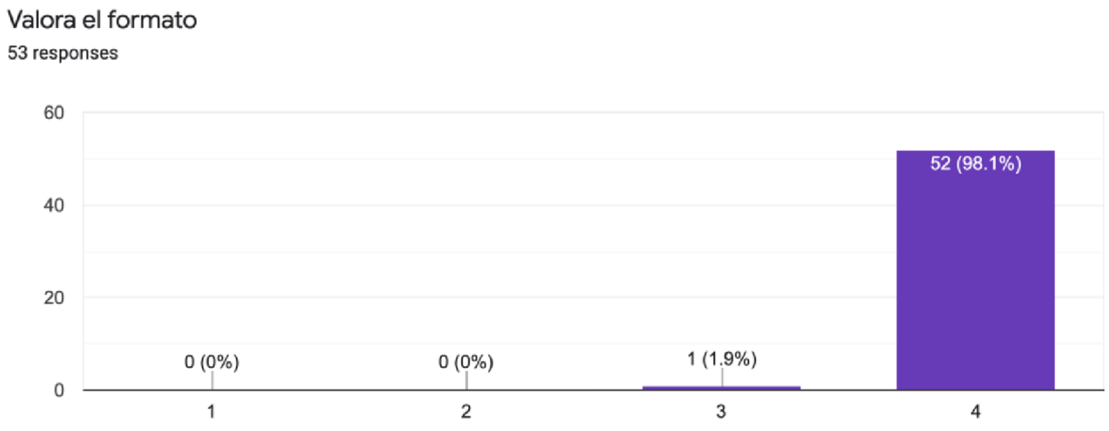

17. Rate the diversity

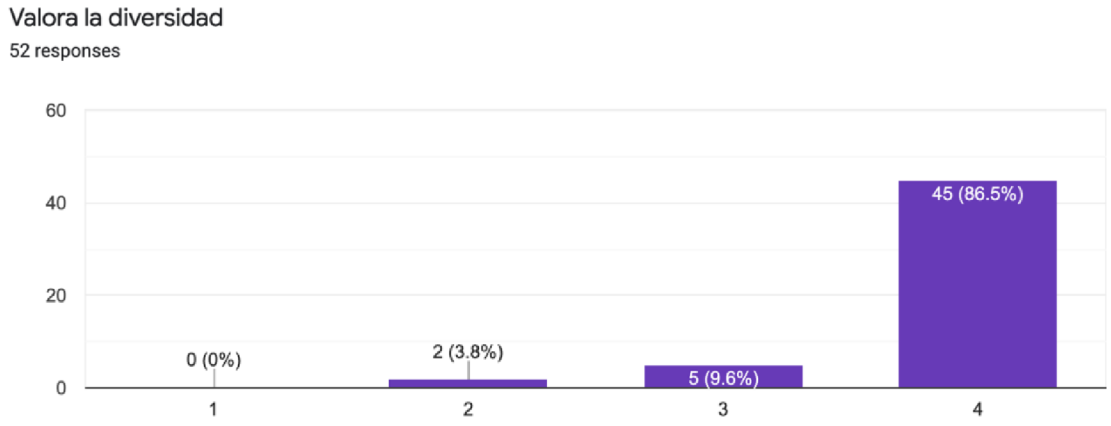

19. Value the proportion of people involved in each session

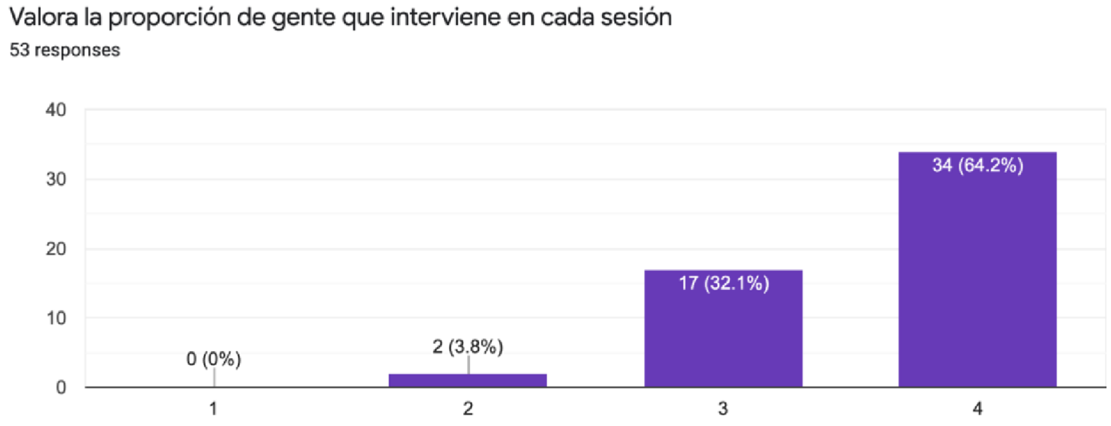

21. Evaluate the time spent in the debate

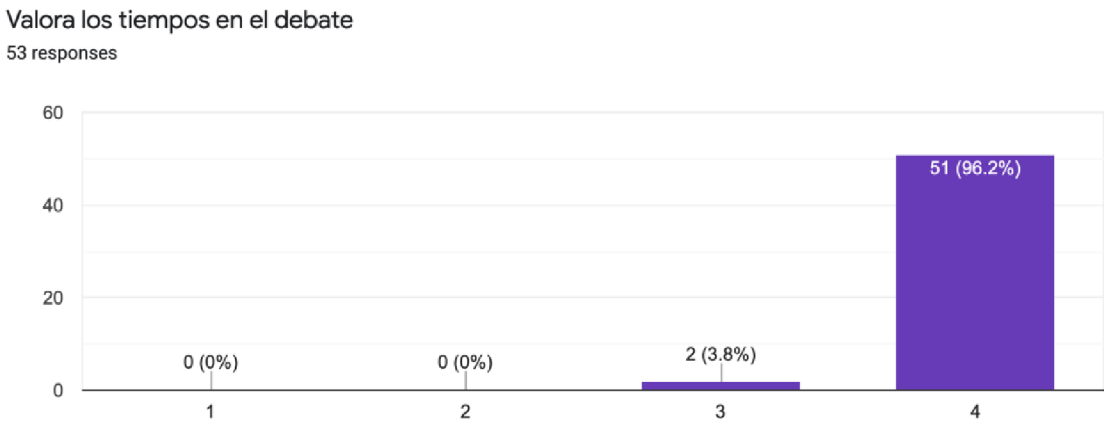

23. Rate the selection of the films

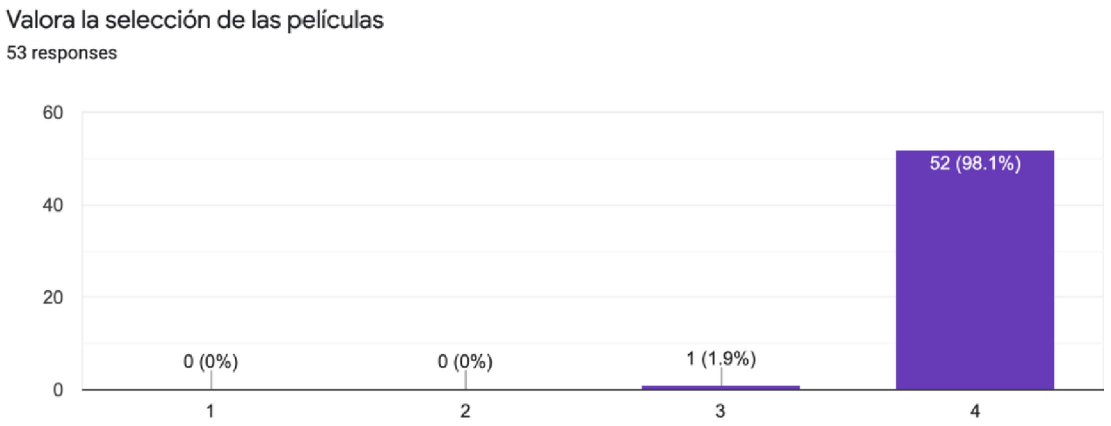

25. Assess the intellectual level of the debates

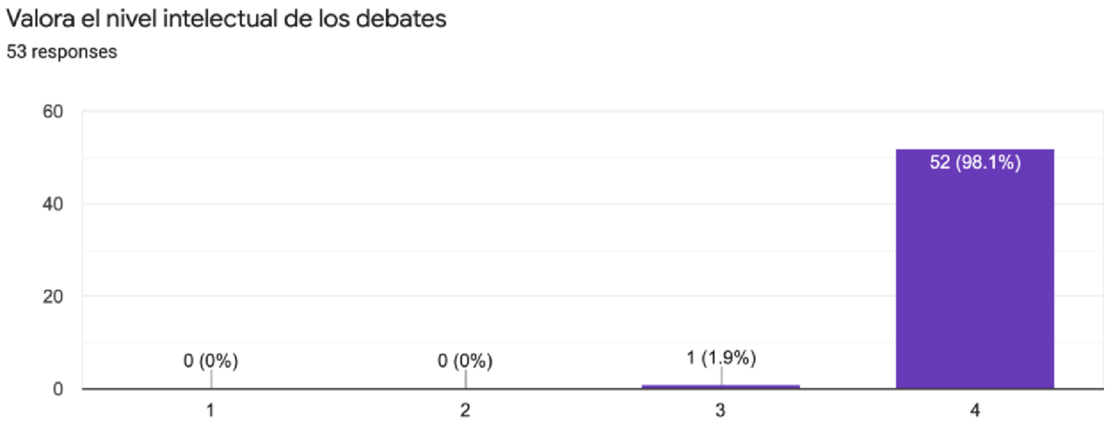

27. Value the knowledge that complements the debate

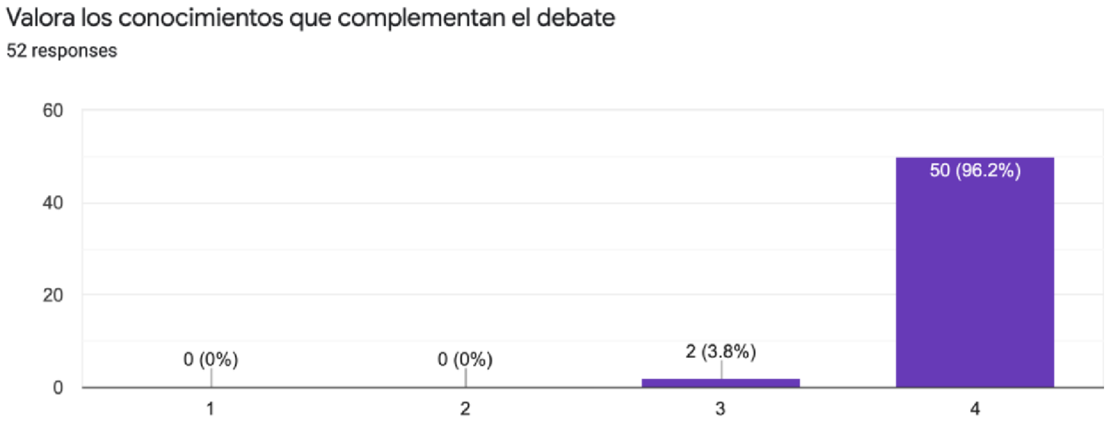

29. Value the freedom to express an opinion in the debate

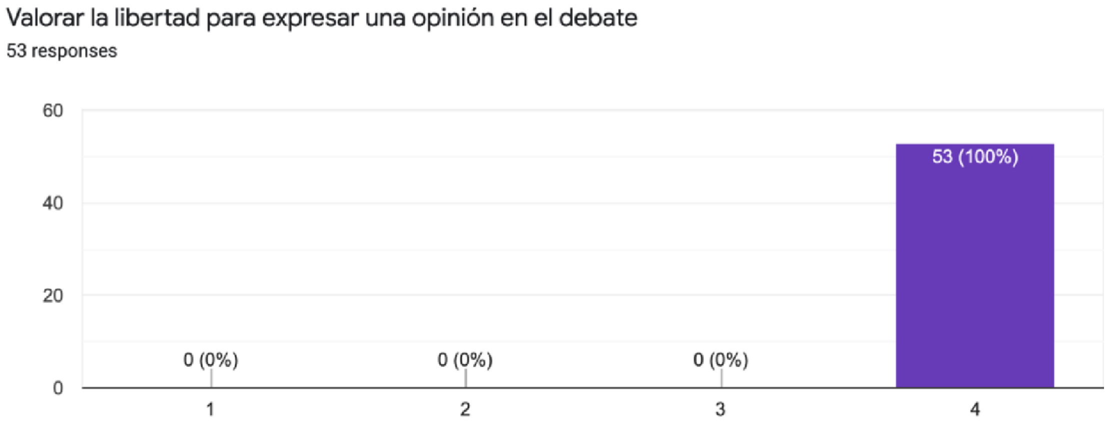

31. Value the frequency with which all contributions are respected

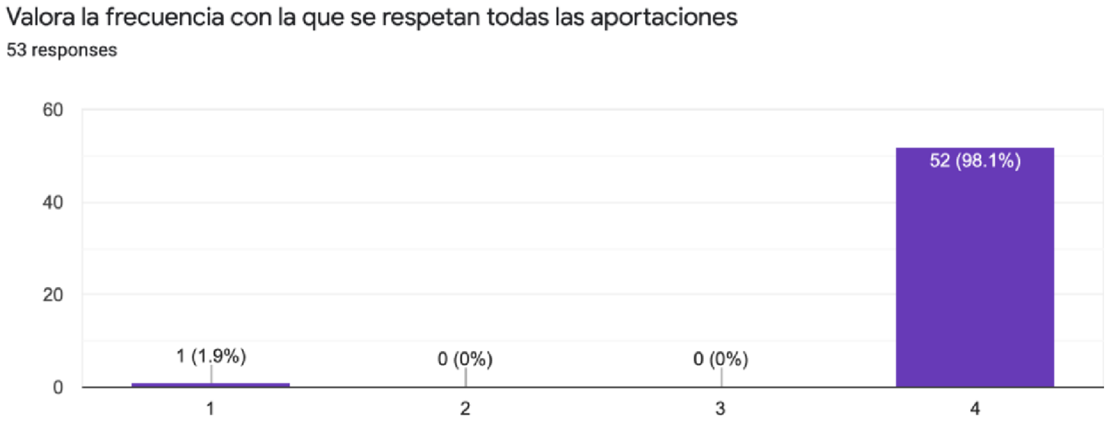

38. Rate how much participating in the Dialogic Gathering of Films has helped you at a professional level

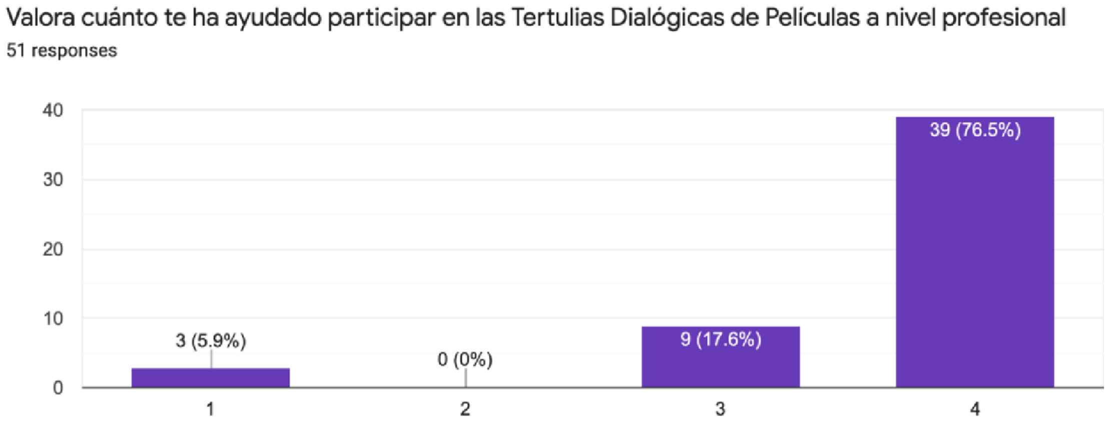

41. Rate how much you think participating in Dialogic Gathering of Films can help you professionally

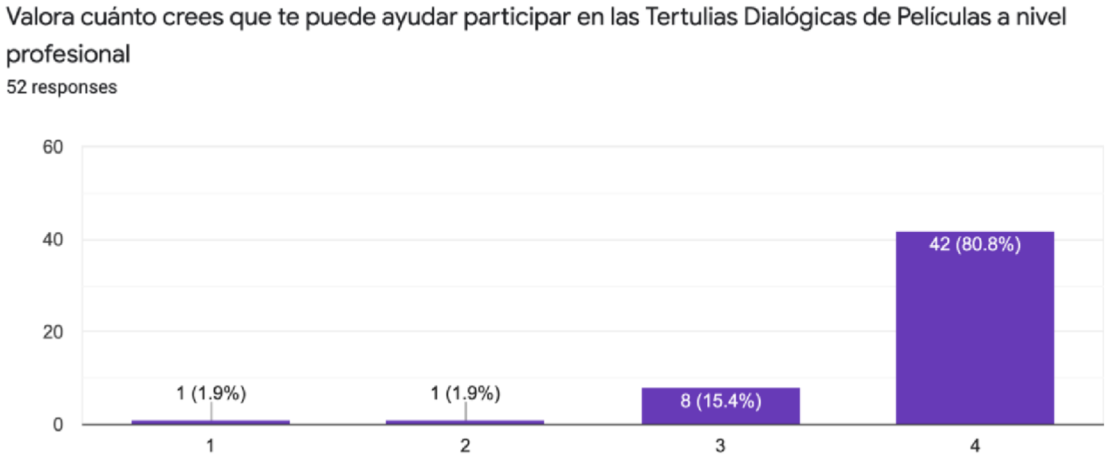

42. Rate how much has participating in the Dialogic Gathering of Films helped you on a personal level?

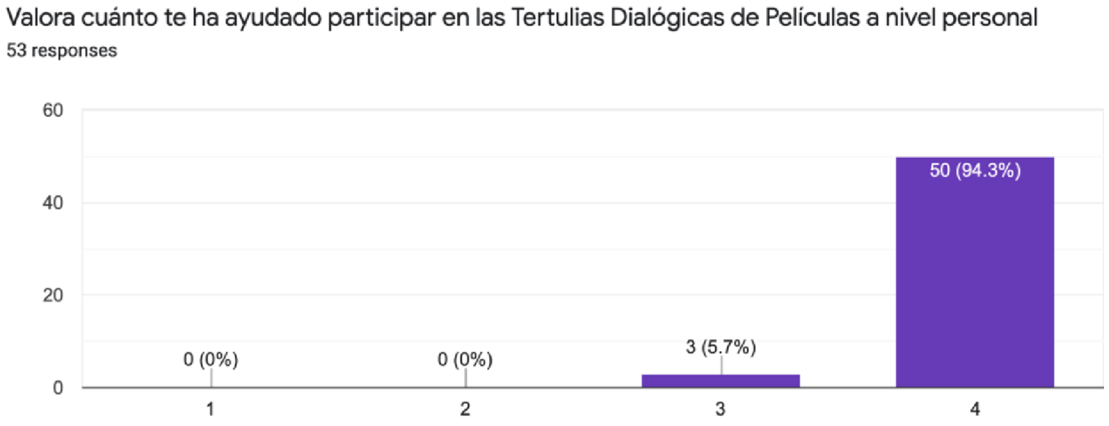

45. Rate how much has participating in the Dialogical Gathering of Films helped you to deal with the situation generated by the COVID-19 pandemic

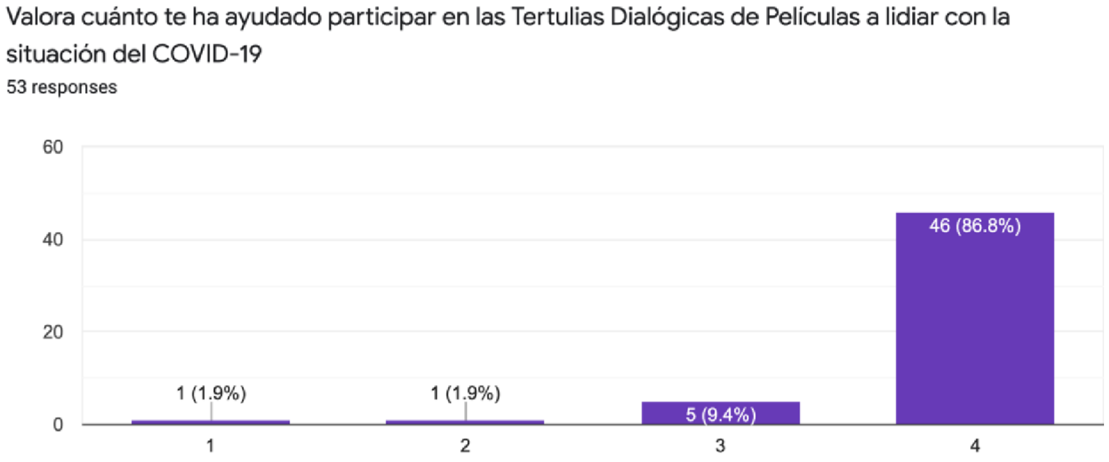

47. Rate the extent to what, in comparison with other activities, you have been encouraged by the Dialogic Gathering of Films to work against COVID-19

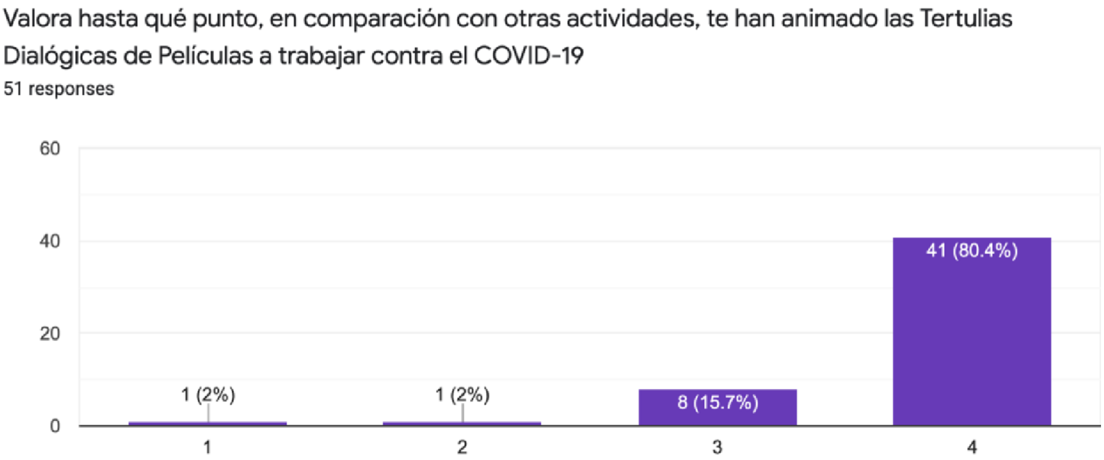

49. Rate how much the Dialogic Gathering of Films benefits you.

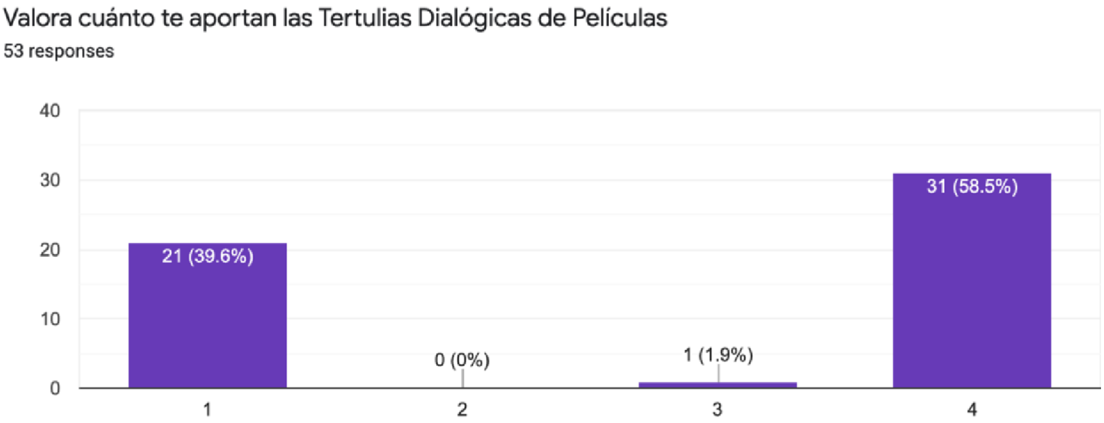

Supplement: S1 Dataset — (PDF) [file pone.0254132.s003.pdf]
