# Supplementary material for: Dialogic gathering of films. Promoting meaningful online interactions during COVID-19 confinement
Source: PLoS One. 2021 Jul 9;16(7):e0254132. doi: 10.1371/journal.pone.0254132 (PMC8270149; doi:10.1371/journal.pone.0254132)
Supplement: Attachment — Submitted filename: ResponsetoReviewers.pdf [file pone.0254132.s004.pdf]

RESPONSE TO REVIEWERS

We thank the reviewers and the editor for the suggestions made and the opportunity to improve the manuscript. We have also edited the manuscript with AJE services.

In what follows we provide a detailed table in which we have addressed the reviewer's and editor's comments citing the line number (when necessary). The changes are highlighted in yellow in the file labelled "Revised Manuscript with Track Changes".

EDITOR RESPONSES

Following the editor indications we have revised the style requirements, we have edited the manuscript with AJE services, we have incorporated Supporting Information (questionnaires and data set) and we have included the Tittle page within the manuscript as required. In the following table we address other responses to specific comments from editor.

EDITOR COMMENT	RESPONSE
In the revised version of the paper please consider a text proofreading.	We have edited the document with AJE. The certificate it is attached.
Please take a close look to the tables listed in the paper - please replace Table X in row 182 with its associated number.	Thank you for this observation. All the tables have been revised and renumbered when necessary.
Please provide more information related the Communicative Methodology you have used in the paper and provide some arguments for this choice.	Arguments for the choice of Communicative Methodology have been included in lines 105-122.
In the questionnaire provided as supplementary materials there are a series of descriptive statistics regarding the respondents - please add the summary of this information in the paper, in addition to the information already provided in Table 2.	According with this suggestion, descriptive statistics regarding the respondents (gender, age, country, city of residence and professional field) are presented in Tables 2, 3 and 4.
Please add a validation for the questions for which the answers have been gathered using a Likert scale.	Thank you for this comment that allows us to explain more about the methodology process. As we are analyzing a new reality

	not studied before, it was not possible to find a validated existent questionnaire useful for study the DGF. Therefore, researchers created a new questionnaire. The questionnaire was elaborated and validated following the dialogic design steps from the communicative methodology. This is explained in lines 217- 232.

<u>Reviewers' Responses:</u>

Reviewer #1:

**Reviewer 1 comment**	**Response**
I like the introduction of the topic. You provide a nice detailed explanation for your concept. Your method is clear and easy to follow. Your results section does not include an explanation of how the data was analyzed and there is no report of your inter-rater reliability. This is an essential part for qualitative research.	The process of qualitative data analysis is now explained in section Data Analysis (lines 249-274), including the validation of the qualitative findings.
I like the conclusions made and examples provided to support them. However, you are missing an important piece to show that there is consistency between those evaluating the survey answers and the conclusions made. I cannot recommend this article until this information is included.	According to the different comments of reviewers we have separated Conclusions into two sections “3. Discussion” and “4. Conclusions, limitations and further research”. In both sections we argue about the links among the literature review, the results and the conclusions made.

Reviewer #2:

**Reviewer 2 comment**	**Response**
This research covers a relevant topic of research given the ongoing COVID-19 pandemic over more than the past one year. It contributes to the literature to understand coping mechanisms to address loneliness and isolation resulting from COVID.<br>Overall, there are many grammatical errors across the manuscript that need to be address. This affects readability and meaning in places. Suggest using active voice over passive where possible and write the results in past tense. The paper moves between past and present tense. These need to be thoroughly revised.	We have edited the document with AJE. The certificate it is attached
Regarding the structure of the manuscript: Parts of introduction can move to the methods section and parts of methods can be moved to the results or removed where repeated, specific comments provided below.	Following these indications, we have moved some parts of the introduction to the methods section and parts of methods to the results section. Thank you for this useful comment, with this changes the article is clearer.
Suggest having a separate section for conclusion and implications, at present it sits under Discussion. Please highlight any possible Limitations of the study.	According to the different comments of the reviewers, we have separated the Conclusions into two sections "3. Discussion" and "4. Conclusions, limitations and further research". In section 4 we have highlighted the limitations of the study linking it to the possibilities for further research.

Introduction

**Reviewer 2 comment**	**Response**

Line 62, p. 3. Correct the spelling of 'South Korea'.	This has been corrected, thank you.
Line 80, p. 4. under 'Dialogue of Gathering films', please elaborate on the 'vulnerable groups' mentioned.	We have further elaborated around the concept of "vulnerable groups" in lines 86-87.
Line 99, p. 5. Please specify the 'participant needs' or move this part to the methods section.	*Participants' needs* clarification has been included in lines 142-143 and all the part has been moved to the methods section.
Under section 'Dialogue of Gathering films', a lot of it can move to the methods section.	We have moved this information to the methods section.

Methods

**Reviewer 2 comment**	**Response**
Suggest reducing the length of the methods.	We have moved information from methods section to results section reducing the length of methods. At the same time, and following other revisions received, we have also moved parts from the introduction to the methods section and included more explanation in relation to methods section, as was requested in the review process. Therefore, the methods section has not been substantially reduced.
Section 2.1. 'Running of the DGF and discussion resulted from each film' please change to past tense for uniformity.	We have edited this section.
Last paragraph on p.9 and first paragraph is more relevant to the results section, suggest moving it to the results section.	Thank you for your suggestion, we have moved this paragraph to the results section.
Line 195-197, p. 12. Suggest breaking the sentence or rephrase for readability.	We have edited the document rephrasing these lines.

Line 209, p. 12. Suggest rephrasing for clarity to, 'Majority of participants joined from Spain while 4 participants participated from UK, Brazil and the USA.'	We have rephrased these lines as suggested. Thank you
Line 211, p.12, suggest replacing the word 'fields' to 'backgrounds'.	We have replaced this word as you suggested.
Table 3. can include demographic profile of participants such as sex of participants (Male/Female), background and other details, if available, as these are discussed in the Discussion. Please provide details of facilitators – who were they, how were they identified.	Demographic profile of participants (male/female; age; country and place of residence, nationality and professional field) are now included in Tables 3, 4 and 5 and in lines 198-204; 207-210 and 212-214.
Last paragraph p. 16, can be moved to the results or removed as it is already mentioned in the results section.	This paragraph has been removed and some information has been incorporated at the beginning of the results section.

Results

**Reviewer 2 comment**	**Response**
Please provide some detail of the participant whose data are shared verbatim as quotes, this could include sex and age of participant in bracket next to the data quote.	We have included sex and age in brackets at the end of each verbatim quote. Thank you for this suggestion that allows to present the results more accurately.
Change the percentages provided to whole rather than in points, e.g. 94.2% can be 94% and 94.6% can be 95%.	We have changed the percentages according to this comment.
The last data quote on p.24 is in italics, while the one above is not. Kindly revise for	We have removed italics and revised uniformity of quotes.

uniformity.	

Discussion

Reviewer 2 comment	Response
The Discussion section at this stage requires more detail and needs to be contextualised within the wider literature. Suggest elaborating discussion section on the positive impact of DGF on loneliness and helping participants professionally	According to the different comments of the reviewers, we have separated Conclusions in two sections: “Discussion” and “Conclusions, limitations and further research”. Positive impact on loneliness and helping participants professionally have been included.
